# Pancreatitis cytosorbents (CytoSorb) inflammatory cytokine removal: A Prospective Study (PACIFIC)

**DOI:** 10.1097/MD.0000000000013044

**Published:** 2019-01-25

**Authors:** Wolfgang Huber, Hana Algül, Tobias Lahmer, Ulrich Mayr, Miriam Lehmann, Roland M. Schmid, Andreas Faltlhauser

**Affiliations:** a Medizinische Klinik und Poliklinik II, Klinikum rechts der Isar; Technische Universität, München, Ismaninger Straße 22, D-81675 München; b Medizinische Klinik I, Kliniken Nordoberpfalz, Klinikum, Weiden, Germany.

**Keywords:** acute pancreatitis, cytokine elimination, CytoSorb, hemodynamic monitoring, hemoperfusion, multi organ failure, severe acute pancreatitis, systemic inflammation syndrome, transpulmonary thermodilution, vasopressor dependency index

## Abstract

**Background:**

Acute pancreatitis (AP) usually has a mild course with a mortality rate below 1%. However, around 10% of patients develop severe AP (SAP) involving extra-pancreatic tissues and other organ systems. The mortality of SAP is around 42%. The outcome of SAP is closely related to the development of systemic inflammation and consecutive organ failures. Most current therapies including fluid resuscitation, antimicrobial therapy, drainage procedures, and endoscopic management of complications are symptomatic rather than causative approaches, except sphincterotomy for gallstone pancreatitis. Regarding the high mortality of SAP and its close association with systemic inflammation, extracorporeal removal of inflammatory mediators is an appealing approach. Several recent studies have demonstrated that the CytoSorb adsorber effectively eliminates inflammatory cytokines, such as IL-1ß, IL-6, IL-8, IL-10, and TNF-alpha. Some of these trials suggested that therapy with CytoSorb might improve outcome, including a reduction in the vasopressor dosage and reversal of shock.

Therefore, it is the objective of this study to evaluate the effectiveness of 2 consecutive 24 h-treatments with CytoSorb on hemodynamics in patients with early SAP.

**Methods:**

This study includes patients with early SAP (APACHE-II ≥10) and transpulmonary thermodilution hemodynamic monitoring (PiCCO; EV-1000) within a maximum of seven days from the onset of pain. Eligible patients will be treated with 2 consecutive periods of CytoSorb. A 20%-improvement in the vasopressor dependency index (VDI) - which relates is derived from mean arterial pressure (MAP) and catecholamine dosage - is the primary outcome. In addition to this clinical outcome, there are several laboratory (cytokine levels) and translational endpoints (including multiplex-ELISAs of numerous anti- and pro-inflammatory cytokines/chemokines and DNA analyses). Primary outcome analysis will compare the incidence of the primary endpoint in 30 patients from the intervention group to 60 matched controls with advanced hemodynamic monitoring recruited from recent studies in SAP within the same setting and the same centers.

**Discussion:**

A potential improvement in hemodynamics and/or other outcomes by CytoSorb would provide a new therapeutic option in the early treatment of SAP with a pathophysiological rationale.

**Trial registration:**

This study was registered on March 17, 2017 (ClinicalTrials.gov Identifier: NCT03082469). URL: https://clinicaltrials.gov/ct2/show/NCT03082469.

**Version:**

V_PACIFIC_1.0 September 30, 2018.

## 1 Background

Acute pancreatitis (AP) is an acute inflammatory process of the pancreas.^[1–4]^ In about 90% of patients, AP has a mild course with inflammation localized to the pancreatic gland. Mild AP has a mortality rate below 1%.^[1]^ The remaining 10% of patients develop severe AP (SAP) involving extra-pancreatic tissues and other organ systems. The outcome of SAP is closely related to the development of systemic inflammatory response syndrome (SIRS) and consecutive multiple organ failures (MOF). The mortality of SAP is around 42%.^[5]^

*Diagnosis* of AP is easily established based on the presence of at least 2 of the 3 criteria of typical epigastric pain with radiation to the back, at least 3-fold elevation of serum lipase or amylase, and evidence of AP on abdominal ultrasound or computed tomography (CT). By contrast, *therapy* for SAP is cumbersome and limited due to the lack of causative therapies apart from the removal of bile duct stones. Consequently, the therapy for SAP is predominantly symptomatic. Optimized fluid resuscitation is the cornerstone of these symptomatic therapeutic approaches, since early volume depletion and hemo-concentration have been repeatedly associated with poor outcome. In addition, early goal-directed resuscitation based on transpulmonary thermodilution (TPTD) might potentially improve outcome in SAP.^[4,6,7]^

Severity and mortality of AP are 2-peaked, with an early peak due to overwhelming SIRS and a second peak after 2 to 4 weeks, predominantly due to septic complications. With regard to the marked inflammatory response (“cytokine storm”) several attempts to prevent or limit inflammation have been investigated. However, none of the medical concepts including antioxidants,^[8]^ platelet-activating antagonists,^[9]^ antibodies against mediators of inflammation such as tumor necrosis factor alpha, or non-steroidal anti-inflammatory drugs have been shown to improve outcome in large clinical trials. This might be related to the protracted intervention aimed at limiting SIRS in a fulminant disease with already established “cytokine storm”. In addition to the delayed onset of action of medical immune-modulators, these drugs also carry the risk of side-effects, and may further impair organ function.

In this scenario, blood-purification with hemofiltration and/or hemo-adsorption with immediate elimination of cytokines may be a promising alternative.^[10]^

Several recent studies have investigated the CytoSorb (CytoSorbents Corporation, Monmouth Junction, NJ) cytokine adsorber.^[10–12]^ CytoSorb is a biocompatible adsorber approved for use in patients with elevated inflammatory mediators, with absorption rates for inflammatory cytokines, such as IL-1ß, IL-6, IL-8, IL-10, and TNF-alpha.^[11,13]^ Several studies, predominantly in septic patients, suggest that effective elimination of these mediators may be associated with improved outcome, including reduction in the vasopressor dosage and reversal of shock.^[11]^

### 1.1 Aim, design and setting of the study

It is the objective of the PACIFIC-study to investigate if early administration of CytoSorb in patients with SAP substantially improves hemodynamic characteristics including vasopressor dosage and cardiac power index (CPI).

The PACIFIC-trial is designed as a prospective multi-center case-control study.

The study will be performed in intensive care units (ICU) experienced in the use of CytoSorb and capable of combined TPTD and pulse contour analysis (PCA) monitoring.

All patients will be monitored by PiCCO (Pulsion Medical systems SA, Feldkirchen, Germany) or EV-1000 (Edwards Lifesciences, Irvine, CA).

The matched controls will be recruited from patients with SAP treated in a comparable setting including TPTD-monitoring, but not treatment with CytoSorb or any other cytokine adsorbing devices.

### 1.2 Characteristics of participants and description of materials

#### 1.2.1 Inclusion and exclusion criteria

Patients with SAP and an APACHE-II-score ≥10 are eligible for 7 days after the onset of pain. The main inclusion criteria (see Table 1) are:

- proven AP;
- within 7 days of the onset of pain;
- in combination with criteria associated with increased severity and poor outcome of AP.

**Table 1:**
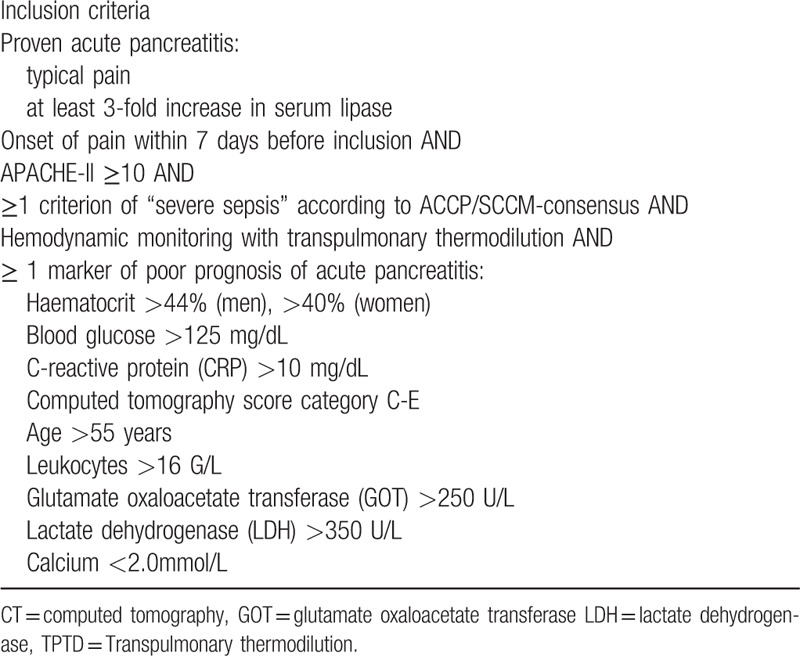
Inclusion criteria.

Inclusion is limited to a maximum of 7 days after the onset of pain. This period is considered as the early phase of SAP, including the first peak of inflammation due to a SIRS.

Patients can only be included if they are under hemodynamic monitoring with TPTD irrespective of the study.

With regard to the representativeness of the data, exclusion criteria are limited in number, and they are predominantly related to ethical reasons (Table 2).

**Table 2:**
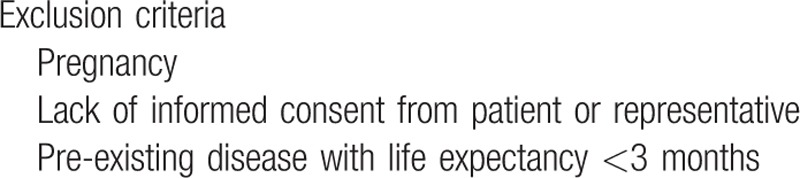
Exclusion criteria.

#### 1.2.2 Intervention

A total of 30 patients will be treated with 2 consecutive 24 h-treatments of cytokine adsorption with the CytoSorb-device. The device will be changed after the first 24 h-treatment period. As appropriate, the CytoSorbents (CytoSorb)-cartridge will be used without additional extracorporeal devices in a hemoperfusion modality or combined with continuous veno-venous hemo (dia)filtration (CVVH (D)F). According to the manufacturer's recommendation the target blood flow is 150 (-200) mL/min. It should not fall below a limit of 120 mL/min.

Anticoagulation will be performed in analogy to renal replacement therapy and at the discretion of the treating physician. In case of heparin anticoagulation, a partial thromboplastin time (PTT) of 50 to 60 s is recommended according to the manufacturer CytoSorbents.

Since the CytoSorb-device is capable of eliminating certain antibiotics, additional dosages will be given according to the manufacturer's recommendations.^[12]^ In case of carbapenem treatment during CytoSorb treatment, an antibiotic dosage in the upper normal range without additional applications is recommended.

#### 1.2.3 Outcomes

Primary and major secondary outcomes are given in Table 3.

**Table 3:**
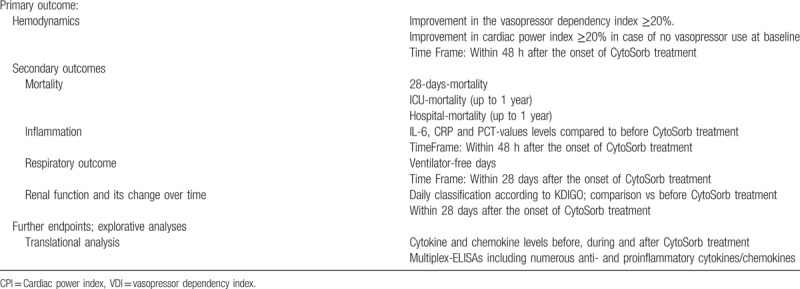
Primary and secondary endpoints.

The primary endpoint is defined as an improvement of hemodynamics with a decrease in the vasopressor dependency index (VDI) of at least 20% within 48 h compared to the baseline measurement immediately before the onset of therapy with CytoSorbents (CytoSorb). For patients without vasopressor therapy at baseline, the primary endpoint is defined as an improvement in CPI of at least 20%.

The VDI is defined as the ratio of the inotropic score divided by mean arterial pressure (MAP):

VDI = inotropic score / MAP

The inotropic score is defined as (dopamine dose_1) + (dobutamine dose∗1) + (adrenaline dose∗100) + (noradrenaline dose∗100) + (phenylephrine dose∗100). All doses are given as μg/kg/min.

TPTD will be performed at baseline as well as after 6, 8, 12, 16, 24, 30, 32, 36, and 48 h after the initiation of blood purification.

Outcome analysis will be based on a comparison of the incidence of the primary endpoint in 30 intervention patients compared to 60 matched controls with advanced hemodynamic monitoring recruited from recent studies in SAP within the same setting and the same centers. To account for different numbers of TPTD measurements in the controls (usually 2–3 measurements within 24 h), the primary outcome analysis will be performed based on the measurements at baseline as well as after 12, 24, 36, and 48 h after the initiation of CytoSorb-therapy.

APACHE-II-score on inclusion and baseline VDI are the main criteria for matching.

Secondary analyses will include laboratory (cytokine levels) and translational endpoints (including multiplex-ELISAs of numerous anti- and pro-inflammatory cytokines/chemokines and DNA analyses).

#### 1.2.4 Pre-defined subgroups

Pre-defined subgroup analyses will be performed for the subgroups of patients with alcoholic vs non-alcoholic etiology, for patients with biliary vs non-biliary etiology, for patients with older age (defined as age ≥65 years vs age <65 years) and the subgroups with and without vasopressor use at baseline.

Table 4 summarises the schedule of events.

**Table 4:**
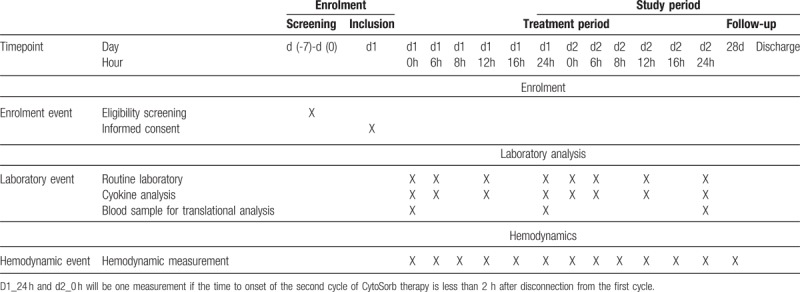
Schedule of events.

### 1.3 Statistics

The incidences of the primary endpoint and all dichotomous secondary endpoints will be compared using the Chi-Square test or Fisher exact test as appropriate.

All comparisons of continuous endpoints within groups and between groups will be performed using *t* test or Wilcoxon-test for paired or unpaired samples as appropriate.

A p-value of *P*<.05 is considered as significant.

Missing values over time will be replaced using “last value carried forward”.

#### 1.3.1 Power calculation

Power calculation is based on the assumption that the primary endpoint is fulfilled in 67% of the 30 patients in the intervention-group and in 33% of the 60 matched controls. This results in a statistical power of 87.8% (https://www.dssresearch.com/KnowledgeCenter/toolkitcalculators/statisticalpowercalculators.aspx).

### 1.4 Data management

Data including baseline data and medical history as well as side effects will be documented in a pre-defined case report form. Data will be transferred to a central database at the center of the principal investigator.

Patients will be followed up until discharge or death.

## 2 Discussion

AP is a common diagnosis worldwide. More than 240,000 cases per year are reported in the United States alone. The outcome of AP largely depends on the severity of the disease. Still, it remains a challenge to accurately assess the severity of AP during its early stages. Although recent studies have deepened our understanding of the pathophysiology, most current therapies remain directed at relief of symptoms rather than improving outcome, apart from sphincterotomy for gallstone pancreatitis. While surgery for AP is mainly reserved for severe complications, other therapies such as fluid resuscitation,^[6,14]^ antimicrobial therapy,^[15]^ drainage procedures,^[16]^ and endoscopic management of complications define the current standard of care.

Due to the lack of *causative* therapeutic approaches, the concept to limit local and systemic inflammation is appealing. Acinar cell damage leads to a local inflammatory response but inflammatory mediators also enter the general circulation. These mediators play a key role in AP and the resultant multiple organ dysfunction or MOF syndrome, which is the primary cause of death in patients with SAP. The severity of an attack of AP appears to be determined by the magnitude of the resultant systemic inflammatory response. While excessive stimulation of the immune system accounts for the early systemic complication, immune paralysis, also termed compensatory anti-inflammatory response syndrome, contributes to the local and septic problems in the late phase.

However, several pharmacological approaches including anti-oxidative strategies with vitamin C, selenium and n-acetylcysteine^[8]^ as well as platelet factor antagonists^[9]^ have not resulted in improved outcome for patients with SAP. Compared to these more specific pharmacological approaches, the extracorporeal removal of large amounts of cytokines might be a more immediate and effective approach, in particular in the *early* phase of SAP.

Therefore, this study restricts inclusion to a maximum of 7 days after the onset of pain. Also considering the resources required and the imminent risks of an extracorporeal circuit with anticoagulation, only patients with SAP and a high risk of organ failure will be included. The main criterion for severity is an APACHE-II-score ≥10. Baseline values and changes of APACHE-II are strongly associated with severity and outcome of AP.^[17]^

With regard to the complexity of numerous diagnostic and therapeutic approaches in SAP, the impact of single measures such as 2-day extracorporeal cytokine elimination should not be overestimated. Necessarily, this has to be considered for the study design, in particular for the primary endpoint and the power calculation. Therefore, we chose changes in the VDI as the primary endpoint. VDI has been repeatedly used as an endpoint in studies focused on extracorporeal cytokine and toxin elimination.^[12,18]^

Patients similar to the patients selected according to the inclusion criteria of PACIFIC have been treated by most PACIFIC study centers during the randomized controlled EAGLE-trial (https://clinicaltrials.gov/ct2/show/NCT00894907). The EAGLE-study investigates the impact of TPTD guided volume resuscitation on outcome. Half of the intended number of 182 patients of the EAGLE-study will be under hemodynamic monitoring with the PiCCO-device. Since the inclusion criteria and the clinical setting are nearly identical as for PACIFIC, data from patients from the EAGLE-study with PiCCO-monitoring will be a major source for the allocation of matched controls.

In addition to the primary endpoint and several standard secondary outcomes such as organ failures and mortality, the PACIFIC-trial will analyze several measures of inflammation at baseline and over time. The importance of pro-inflammatory cytokines in pancreatitis has been highlighted by a recent study in 84 patients with AP who were screened for known polymorphisms in TNFα, interleukin 1 (IL-1), IL-1 receptor antagonist (IL1RN), IL-6, and IL-10. The study demonstrated that the TNFα −238 AG genotype, but not the TNFα −308 SNP, was associated with organ failure (shock and/or respiratory failure) during AP.^[19,20]^ A very interesting candidate that seems to drive the pathophysiology of MOF during AP in experimental animal models is IL-6. IL-6 not only regulates the acute phase response in the liver, it can also activate a plethora of pro-inflammatory pathways, such as Stat3 and NF-kappaB.^[21]^

Given the importance of cytokines in AP and the mode of action of CytoSorb, the PACIFIC-study will analyze cytokine and chemokine levels before and after the usage of CytoSorb adsorber. Such analysis will include multiplex-ELISAs of numerous anti- and pro-inflammatory cytokines/chemokines. In an explorative part of the study, DNA from the blood of these patients will be extracted for genetic analysis, particularly with respect to SNPs of factors of interest. All these analyses will add significant information to the PACIFIC studies.

### 2.1 Trial status

The study will start recruitment in October 2018. A number of pilot data have been collected between January 2016 and July 2018. These data will be published separately from the PACIFIC study.

## Author contributions

**Conceptualization:** Wolfgang Huber, Hana Algül, Roland M. Schmid, and Andreas Faltlhauser.

**Data curation:** Tobias Lahmer, Ulrich Mayr, and Miriam Lehmann.

**Formal analysis:** Hana Algül, Tobias Lahmer, and Miriam Lehmann.

**Investigation:** Hana Algül, Tobias Lahmer, and Ulrich Mayr.

**Methodology:** Wolfgang Huber, Hana Algül, and Andreas Faltlhauser.

**Project administration:** Wolfgang Huber and Miriam Lehmann.

**Resources:** Wolfgang Huber and Roland M. Schmid.

**Supervision:** Wolfgang Huber and Roland M. Schmid.

**Validation:** Tobias Lahmer.

**Writing – original draft:** Wolfgang Huber and Hana Algül.

**Writing – review & editing:** Wolfgang Huber, Hana Algül, Tobias Lahmer, Ulrich Mayr, Miriam Lehmann, Roland M. Schmid, and Andreas Faltlhauser.

Wolfgang Huber orcid: 0000-0001-9086-7908.
